# Transcriptome Analysis of Soybean Leaf Abscission Identifies Transcriptional Regulators of Organ Polarity and Cell Fate

**DOI:** 10.3389/fpls.2016.00125

**Published:** 2016-02-17

**Authors:** Joonyup Kim, Jinyoung Yang, Ronghui Yang, Richard C. Sicher, Caren Chang, Mark L. Tucker

**Affiliations:** ^1^Soybean Genomics and Improvement Laboratory, Agricultural Research Service, United States Department of AgricultureBeltsville, MD, USA; ^2^Department of Cell Biology and Molecular Genetics, University of MarylandCollege Park, MD, USA; ^3^Crop Systems and Global Change Laboratory, Agricultural Research Service, United States Department of AgricultureBeltsville, MD, USA

**Keywords:** transcription factors, network, abscission, soybean, *Glycine max*, organ polarity, cell fate

## Abstract

Abscission, organ separation, is a developmental process that is modulated by endogenous and environmental factors. To better understand the molecular events underlying the progression of abscission in soybean, an agriculturally important legume, we performed RNA sequencing (RNA-seq) of RNA isolated from the leaf abscission zones (LAZ) and petioles (Non-AZ, NAZ) after treating stem/petiole explants with ethylene for 0, 12, 24, 48, and 72 h. As expected, expression of several families of cell wall modifying enzymes and many pathogenesis-related (PR) genes specifically increased in the LAZ as abscission progressed. Here, we focus on the 5,206 soybean genes we identified as encoding transcription factors (TFs). Of the 5,206 TFs, 1,088 were differentially up- or down-regulated more than eight-fold in the LAZ over time, and, within this group, 188 of the TFs were differentially regulated more than eight-fold in the LAZ relative to the NAZ. These 188 abscission-specific TFs include several TFs containing domains for homeobox, MYB, Zinc finger, bHLH, AP2, NAC, WRKY, YABBY, and auxin-related motifs. To discover the connectivity among the TFs and highlight developmental processes that support organ separation, the 188 abscission-specific TFs were then clustered based on a >four-fold up- or down-regulation in two consecutive time points (i.e., 0 and 12 h, 12 and 24 h, 24 and 48 h, or 48 and 72 h). By requiring a sustained change in expression over two consecutive time intervals and not just one or several time intervals, we could better tie changes in TFs to a particular process or phase of abscission. The greatest number of TFs clustered into the 0 and 12 h group. Transcriptional network analysis for these abscission-specific TFs indicated that most of these TFs are known as key determinants in the maintenance of organ polarity, lateral organ growth, and cell fate. The abscission-specific expression of these TFs prior to the onset of abscission and their functional properties as defined by studies in Arabidopsis indicate that these TFs are involved in defining the separation cells and initiation of separation within the AZ by balancing organ polarity, roles of plant hormones, and cell differentiation.

## Introduction

Cell separation processes take place throughout the life cycle of a plant including root emergence during seed germination, dehiscence of anthers and seedpods, and shedding of organs (abscission; Roberts et al., 2002). Abscission is an active biological process critical to the survival and reproduction of plants (Bleecker and Patterson, 1997; Patterson, 2001; Taylor and Whitelaw, 2001; Lewis et al., 2006; Meir et al., 2010; Basu et al., 2013). Plant hormones and abiotic/biotic stresses activate the process when the organ (e.g., leaf, flower, fruit) has completed its purpose on the main body of a plant or as part of a defense mechanism. Regulated by various endogenous and exogenous signals, abscission occurs within a specialized tissue called the abscission zone (AZ) consisting of small, less vacuolated cells (Addicott, 1982). Recent transcriptome studies of abscission in a diverse set of plants have provided many insights into the regulation and cellular mechanisms used for organ separation (Cai and Lashbrook, 2008; Meir et al., 2010; Nakano et al., 2013; Wang et al., 2013; Zhang et al., 2015). These studies revealed that there are functional categories of genes such as cell wall hydrolytic enzymes (e.g., polygalacturonases and cellulases) that are commonly up-regulated in abscission of different organs in multiple species. However, although some functional categories of genes may be common to many forms of abscission, the signals that initiate their expression may vary. For instance, more than 100 years ago the plant hormone ethylene was discovered to play an important role in abscission; however, although ethylene has been demonstrated to be essential for abscission in tomato (Lanahan et al., 1994; Meir et al., 2010) and soybean (Tucker and Yang, 2012), ethylene is not essential for floral organ abscission in Arabidopsis (Patterson, 2001). Moreover, genetic and biochemical studies of Arabidopsis floral organ abscission identified additional key signaling components in the regulation of organ separation that are independent of ethylene (Butenko et al., 2003; Patterson and Bleecker, 2004; Liljegren et al., 2009; Leslie et al., 2010; Lewis et al., 2010; Burr et al., 2011; Kim et al., 2013; Gubert et al., 2014; Tucker and Kim, 2015).

Based on many years of abscission research, a working model for abscission has been established. The model differentiates abscission into four developmental phases: Phase (1) establishment of the AZ; Phase (2) acquisition of competence to respond to abscission signals; Phase (3) activation of abscission/cell separation; and Phase (4) trans-differentiation between the separating sides of the AZ cells and formation of a protective scar (Bleecker and Patterson, 1997; Patterson, 2001; Lewis et al., 2006; Liljegren, 2012; Kim, 2014; Tucker and Kim, 2015). Although we have learned much from genetic, gene expression and proteomic studies of abscission, the regulation of gene expression in the AZ by transcription factors (TFs) and their regulatory networks are only beginning to be deciphered (Nath et al., 2007; Nakano et al., 2013; Niederhuth et al., 2013; Wang et al., 2013; Ito and Nakano, 2015). In the current study, we conducted RNA-seq using RNA isolated from soybean leaf AZ (LAZ) and non-abscission zone (NAZ) petiole tissues exposed to ethylene for 0, 12, 24, 48, and 72 h. After removal of the leaf blade, which is a source of auxin that inhibits abscission, exposure to ethylene induces and synchronizes abscission in the LAZ culminating in nearly 100% organ separation by 72 h. Moreover, by exposing both LAZ and petioles to a high concentration of ethylene this reduces differential gene expression between the LAZ and NAZ that might otherwise occur due to unequal synthesis of ethylene in the AZ compared to the petiole, which would cause unequal ethylene-induction of senescence and ethylene-associated defense responses (Tucker and Yang, 2012). We focus herein on the regulatory networks that underlie soybean leaf abscission. The objective of our study was to identify transcription factors that potentially regulate genes affecting the second and third phases of abscission, competence to respond to abscission signals and activation of abscission. We identified transcriptional networks in which transcription factors themselves regulate other transcriptional co-regulators that mediate the separation process.

## Materials and methods

### Plant material

**Figure 1:**
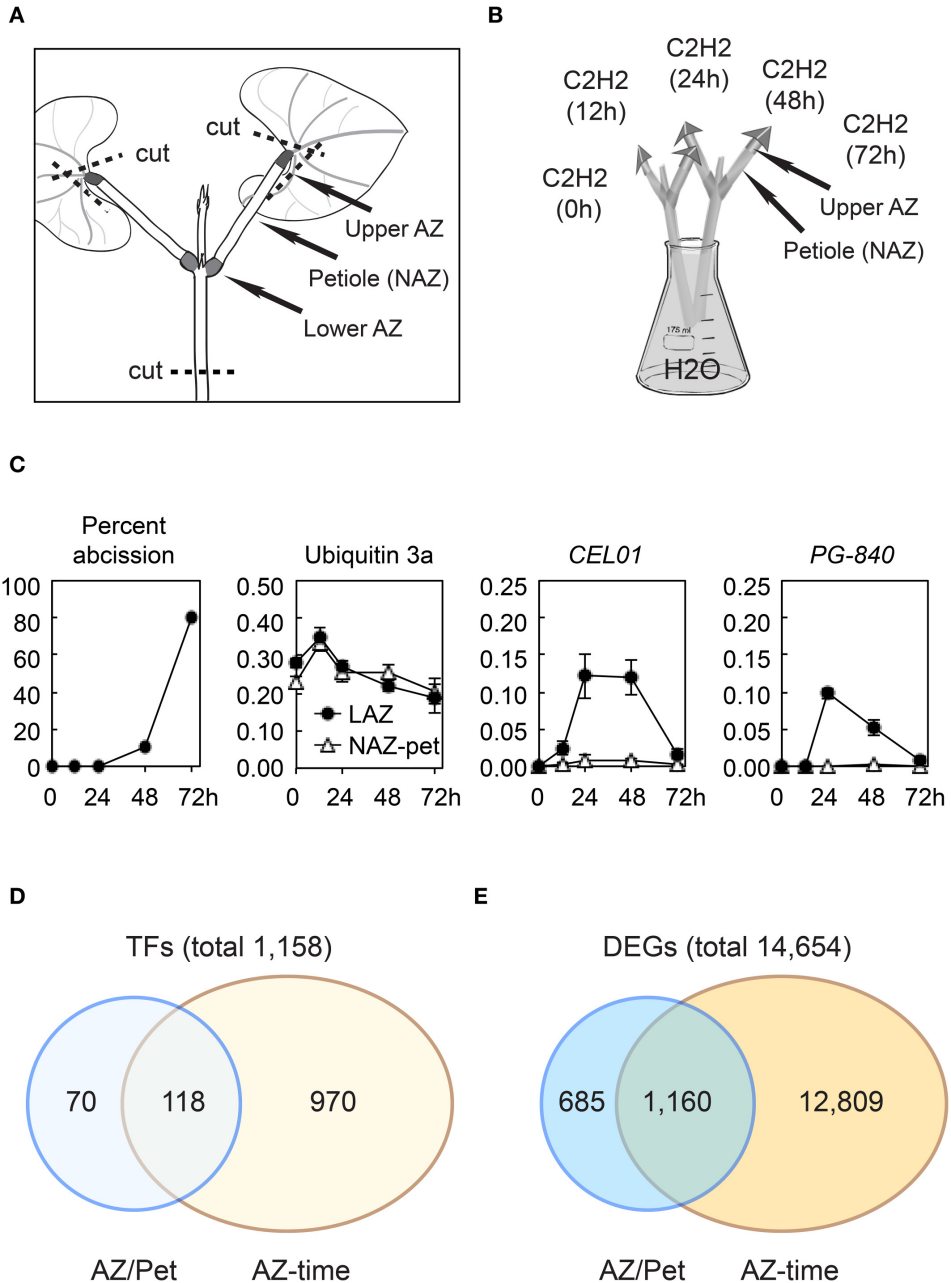
**Soybean leaf abscission system and the overview of transcriptome changes in abscission**. **(A)** Stem-petiole explants were prepared by cutting the leaf blade off leaving an ~5-mm triangular portion of the leaf blade still attached to the petiole and cutting the stem at ~4 cm below the node (dashed line). **(B)** Explants were treated with 25 μL L^−1^ ethylene in air saturated with water. The upper AZ (LAZ) and Non-AZ (NAZ, petiole) were collected at 0, 12, 24, 48, and 72 h. **(C)** Percentile of soybean leaf abscission and plots of RNA-seq results for a constitutive ubiquitin gene *UBI3a* (Glyma20g27950) and two abscission specific marker genes, *CEL01* (Glyma11g02350) and *PG-840* (Glyma20g02840). **(D)** Summary of soybean transcription factors (TFs) differentially regulated more than eight-fold (log_2_ > 3 or < −3, *p* < 0.015) **(E)** Summary of differentially expressed genes (DEGs) in the entire transcriptome that were differentially expressed more than eight-fold (log_2_ >3 or < −3, *p* < 0.015).

Soybean (*Glycine max*, cv. Williams82) seeds were germinated and plants grown in the greenhouse until the primary leaves were fully expanded (19–24 days) and the stem, petioles, and leaves were harvested together by cutting the stem immediately above the soil. Explants were prepared as previously described (Kim et al., 2015) by cutting off the leaf blade leaving about a 5-mm triangular portion of the leaf, and then cutting the stem ~4 cm below the node (Figure 1A). Removing the leaf blade is essential because it removes a source of auxin that inhibits abscission (Addicott, 1982; Meir et al., 2010). Explants were put into Erlenmeyer flasks with water and placed in a dark chamber wherein 25 μL L^−1^ ethylene in air saturated with water was passed through at a flow rate of 2 L min^−1^ (Figure 1B). Treatment with ethylene both accelerates and synchronizes abscission, which is important because AZs and petioles were collected from 20 explants per time point (Addicott, 1982; Tucker et al., 1988). Explants were removed from the chamber after 12, 24, 48, and 72 h, and a 2 mm section of the upper pulvinar AZ (LAZ) at the distal end of the petiole was harvested and flash frozen in liquid nitrogen. In addition, the petiole, non-AZ (NAZ), between the upper and lower AZ was collected and flash frozen (Figure 1B). The upper AZ and petiole were also collected from explants immediately prior to the ethylene treatment, 0 h. To assess the extent of separation at the LAZ, the distal part of the LAZ was gently touched with forceps and, if it fell off the petiole, the LAZ was recorded as having fully abscised (Figure 1C). The AZs at the base of the petioles at the stem interface (lower AZ) were not collected, because the petioles for primary leaves of soybean are rather small and we wanted to avoid incidental collection of a portion of the lateral bud, which would compromise the interpretation of abscission-associated gene expression. LAZ and petioles (NAZ) were collected from 20 explants (two AZs and petioles per explant or 40 LAZ and NAZ total), and the entire process of plant growth, treatment, and collection of LAZ and NAZ was repeated three times.

### RNA sequencing

RNA was isolated from LAZ and NAZ tissue collected after 0, 12, 24, 48, and 72 h after ethylene exposure using a Qiagen RNeasy Mini Kit following the standard protocol (Qiagen, Germantown, MD, USA). Three experimental replications resulted in 30 RNA samples. Further RNA purification, cDNA synthesis and sequencing on an Illumina GAII sequencer were performed at Cornell University, Ithaca, NY, USA as previously described (Zhong et al., 2011; Grassi et al., 2013). The 30 RNA samples were processed, barcoded and run together in a single lane on the GAII sequencer. The raw sequence files have been submitted to the NCBI SRA databases with the study accession SRP050050. On average, each RNA sample produced ~4 million reads. Raw sequences were trimmed to remove ambiguous ends. Using Bowtie (Langmead et al., 2009), ~40,000 (1%) of the reads mapped to ribosomal RNA (rRNA), which were removed from the dataset. Using TopHat (Trapnell et al., 2009), ~90% of the remaining RNA mapped to a predicted data set of soybean transcripts (cds, *G. max* 189 genome assembly) for 54,175 soybean genes. Multiple versions (splice variants) were not taken into account. A single version (usually the last version) was used for alignment. The trimmed sequences (reads) were aligned to the genome assembly and the number of reads aligning with each transcript was normalized to Reads Per Kilobase per Million mapped reads (RPKM) (Mortazavi et al., 2008). For a gene to be counted as expressed in the LAZ or NAZ, we required that the mean RPKM for the three replicates be at least 1.0 or greater in at least one of the treatments. To avoid ratios with a zero in the numerator or denominator, any RPKM of < 0.1 was given the minimal value of 0.1. qPCR was performed as previously described (Tucker et al., 2007) on a few selected genes to confirm that the RNA-seq and RPKM normalization produced the expected expression profile (results not shown).

### Cluster analysis

Ratios for the change in expression over time were obtained by dividing the ethylene-treated time values (12, 24, 48, and 72 h) with corresponding values at 0 h, and ratios for abscission-specificity were obtained by dividing the expression in the LAZ relative to the expression in the petioles (NAZ) at the same time interval. Genes were selected for further analysis based on differential expression greater than eight-fold (log_2_ >3 or < −3, *p* < 0.015) in the LAZ relative to the NAZ (LAZ/NAZ) and also those that changed >eight-fold over time in the LAZ (LAZ at 12/0, 24/0, 48/0, or 72/0 h). These genes and subsets of these genes were then clustered and the uncertainty of the clustering determined using pvclust in the R statistical package (Suzuki and Shimodaira, 2006).

### Gene ontology analyses and generation of transcriptional networks

The Arabidopsis orthologs that best matched the selected soybean TFs and differentially expressed genes (DEGs) were analyzed for Gene Ontology (GO) enrichment and process interactions using BiNGO_Biological_Process (Maere et al., 2005) on Cytoscape v. 3.2.1 using a Benjamini and Hochberg False Discovery Rate (FDR) < 0.05 (5%).

To predict transcriptional networks underlying soybean leaf abscission, we used the Arabidopsis Transcriptional Regulatory Map (ATRM) data set as described by Jin et al. (2015). Details of transcriptional interactions (either activation or repression) are as described in their Figure S1B and Materials and Methods Section (Jin et al., 2015) (http://atrm.cbi.pku.edu.cn/download.php). Using the Arabidopsis TFs that were most similar to the soybean TFs and were also in the ATRM data set (Table 1), transcription networks were generated that reflect interactions that presumably also occur in soybean leaf abscission. Visualizations of the transcriptional networks were generated using Cytoscape v. 3.2.1.

**Table 1:** **List of soybean abscission-specific transcription factors used to generate transcriptional network**.

**Soybean TF (58)**	**Cluster**	**AZ/NAZ**	**TIME/Oh**	**Arabidopsis**	
	**Figure 3**	**O h 12 h 24 h 48 h 72 h**	**12 h 24 h 48 h 72 h**	**TF (40)**	
Glyma02g02630		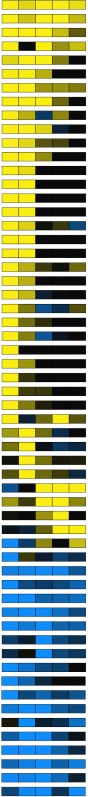	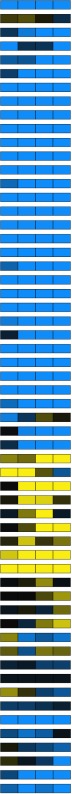	AT3G01470	Homeobox 1, ATHB-1
Glyma17g35951				AT4G36930	SPATULA, SPT AT1G67260 TCP1
Glyma18g51581				AT1G67260	TCP1
Glyma06g45554				AT3G23250	Myb domain protein 15
Glyma02g08241				AT3G54220	SCARECROW, SCR, SGR1, SHOOT GRAVITROPISM 1
Glyma03g34960				AT5G03680	PETAL LOSS, PTL
Glyma03g31530	TF C1			AT4G14550	lndole-3-acetic acid inducible 14, SOLITARY ROOT
Glyma19g34380				AT4G14550	lndole-3-acetic acid inducible 14, SOLITARY ROOT
Glyma02g16071	TF C1			AT3G04730	Indoleacetic acid-induced protein 16
Glyma13g22620				AT2G26580	YAB5, YABBY5
Glyma17g12200	TF C1			AT2G26580	YAB5, YABBY5
Glyma08g28691	TF C1			AT1G67260	TCP1
Glyma18g16390	TF C1			AT3G01470	Homeobox 1, ATHB-1
Glyma03g34710	TF C1			AT5G03790	HOMEOBOX 51, LATE MERISTEM IDENTITY1, LMI1
Glyma05g04260	TF C1			AT2G45190	ABNORMAL FLORAL ORGANS, AFO, FIL,YAB1, YABBY1
Glyma17g14710	TF C1			AT2G45190	ABNORMAL FLORAL ORGANS, AFO, FIL,YAB1, YABBY1
Glyma02g16080	TF C1			AT3G23050	lndole-3-acetic acid 7
Glyma04g10125	TF C1			AT1G23420	INNER NO OUTER, INO
Glyma06g10110	TF C1			AT1G23420	INNER NO OUTER, INO
Glyma19g36100	TF C1			AT2G37260	TRANSPARENT TESTA GLABRA 2, TTG2, WRKY44
Glyma08g39951	TF C1			AT2G43060	ILI1 binding bHLH 1
Glyma07g03840	TF C1			AT3G15540	lndole-3-acetic acid inducible 19
Glyma1Og03720				AT3G23050	lndole-3-acetic acid 7
Glyma1Og27881				AT3G62100	lndole-3-acetic acid inducible 30
Glyma08g40705				AT3G01470	Homeobox 1, ATHB-1
Glyma05g03020				AT1G66350	RGA-Iike 1
Glyma18g45220				AT3G54220	SCARECROW, SCR, SGR1, SHOOT GRAVITROPISM 1
Glyma19g05921				AT1G67260	TCP1
Glyma03g33376				AT2G37260	TRANSPARENT TESTA GLABRA 2, TTG2, WRKY44
Glyma1Og07730				AT5G03680	PETAL LOSS, PTL
Glyma09g33241				AT5G10510	AINTEGUMENTA-Iike 6
Glyma03g19030				AT2G37630	ASYMMETRIC LEAVES 1, MYB91,PHANTASTICA-LIKE 1
Glyma02g40650				AT5G37020	Auxin response factor 8
Glyma15g01960				AT1G79840	GL2, GLABRA 2
Glyma13g37111				AT1G03790	SOMNUS (SOM)
Glyma03g06225				AT4G05100	Myb domain protein 74
Glyma04g03801	TF C3			AT5G42630	ABERRANT TESTA SHAPE, ATS, KAN4, KANADI 4
Glyma06g03901				AT5G42630	ABERRANT TESTA SHAPE, ATS, KAN4, KANADI 4
Glyma16g26291	TF C4			AT3G26744	ICE1, INDUCER OF CBF EXPRESSION 1, SCREAM
Glyma09g29940				AT1G17950	Myb domain protein 52
Glyma03g26520				AT2G44840	Ethylene-responsive element binding factor 13
Glyma14g10830				AT4G26150	CGA1, CYTOKININ-RESPONSIVE GATA1, GATA22, GNL
Glyma12g13710				AT4G28500	NAC PROTEIN 73, SECONDARY WALL-ASSOCIATED
Glyma17g06290				AT5G56860	GATA TRANSCRIPTION FACTOR 21, GNC
Glyma06g21495				AT5G61270	Phytochrome-interacting factor7
Glyma08g02020				AT5G41410	BEL1, BELL 1
Glyma05g21726				AT4G32880	Homeobox gene 8, ATHB8
Glyma18g44030				AT2G38470	WRKY33
Glyma06g44250	TF C5			AT4G28500	NAC PROTEIN 73, SECONDARY WALL-ASSOCIATED
Glyma12g33460	TF C5			AT4G28500	NAC PROTEIN 73, SECONDARY WALL-ASSOCIATED
Glyma1Og28820				AT1G23380	KNOTTED1-Iike homeobox gene 6
Glyma11g04910				AT4G37750	AINTEGUMENTA
Glyma18g04580				AT5G16600	Myb domain protein 43
Glyma10g27860	TF C6			AT4G04450	WRKY42
Glyma18g48730				AT2G44840	Ethylene-responsive element binding factor 13
Glyma05g38530				AT3G26744	ICE1, INDUCER OF CBF EXPRESSION 1, SCREAM
Glyma03g39041				AT1G23380	KNOTTED1-Iike homeobox gene 6
Glyma17g00650				AT2G02450	ANAC034, NAC 35, LONG VEGETATIVE PHASE 1

*Of the 188 abscission-specific soybean transcription factors, 58 soybean TFs matched 40 different Arabidopsis homologs found in the high-confidence ATRM data set. More information on these 40 TFs can be found in Table S5. Expression patterns (Up or Down) and the TF Cluster numbers are as shown in Figure 3. AZINAZ, represents the difference in expression in the two tissues at the indicated time; Time/Oh, represents the change in expression at the indicated time relative to 0 h; yellow, up-regulation; black, neutral; blue, down-regulation*.

## Results and discussion

### Overview of transcriptome changes in soybean leaf abscission

We conducted RNA-seq using RNA isolated from soybean leaf AZ (LAZ) and non-abscission zone (NAZ) petiole tissues exposed to ethylene for 0, 12, 24, 48, and 72 h. The RNA-seq expression results for all 54,175 soybean genes can be found at http://sgil.ba.ars.usda.gov/mtucker/Public/Tucker.html. To validate our RNA-seq data, the isolated RNA was used to perform qPCR for several marker genes known to be specifically expressed in the AZ (e.g., cellulase, polygalacturonase, etc; Tucker et al., 1988; Kalaitzis et al., 1999; Kim and Patterson, 2006; Kim et al., 2006; results not shown). Additionally, when the RNA-seq data was plotted for a constitutively expressed ubiquitin and differentially expressed cellulase and polygalacturonase that correlate with cell wall loosening and cell separation, the expression patterns were as previously reported (Tucker et al., 2007; Figure 1C and Table S1).

For a gene to be scored as expressed, we required that a gene have a minimum RPKM value >1 in either the LAZ or petiole at any time interval between 0 and 72 h of ethylene treatment. Using this criterion, we selected 37,572 of the 54,175 soybean genes in the *G. max* 189 genome assembly as being expressed in our tissue collections. Based on the predicted Gene Ontology (GO) biological process and cellular component for the most similar gene identified in Arabidopsis, we selected 5,206 genes in the soybean genome as having transcriptional activity, i.e., transcription factors (TFs) (http://sgil.ba.ars.usda.gov/mtucker/Public/Tucker.html). Within these 5,206 TFs, 3,593 TFs were expressed in the LAZ and/or petiole. To identify transcriptional dynamics associated with organ separation, we narrowed down the TFs for further analysis by selecting only genes that were differentially expressed more than eight-fold (log_2_ > 3 or < −3, *p* < 0.015) in the LAZ relative to the NAZ (LAZ/NAZ) and also those that changed >eight-fold over time in the LAZ (LAZ at 12/0, 24/0, 48/0, or 72/0 h; Figure 1D and Table S2). In addition, we then limited the selection of TFs by eliminating genes having transcriptional activities that are associated with DNA methylation or demethylation, e.g., Glyma20g32960 (At2g36490, Demeter-like 1) and genes with DNA-directed RNA polymerase activity, e.g., Glyma18g17166 (At1g60620, RNA-polymerase I subunit 43). After excluding these genes, there were 1,158 TFs with a more than eight-fold differential expression pattern (Figure 1D). This selection includes genes that are more highly expressed in the petiole than the LAZ (log_2_ < −3). Although it is reasonable to conclude that TF gene expression that is higher in the petiole than the LAZ is more closely tied to processes specific to the petiole, it is also possible that the lack of a TF (e.g., transcriptional repressor) in the AZ could be important to LAZ gene expression and abscission. Therefore, TFs whose expression was lower in the LAZ than the NAZ (log_2_ < −3) were included in the gene clustering and subsequent analyses. However, rather than unnecessarily complicate the discussion of gene expression, both significantly higher and lower expression in the LAZ will be referred to as simply abscission-specific unless it is relevant to the discussion. Thus, within this eight-fold subgrouping, 188 TFs were found to be abscission-specific, and, among these, 118 TFs changed more than eight-fold over time and were also abscission-specific (Figure 1D and Table S2).

Because these differentially expressed TFs presumably regulate downstream events in organ separation and these target processes are also of interest, we selected genes of all types in the entire transcriptome that displayed a more than eight-fold change in expression, i.e., differentially expressed genes (DEGs). When the same criterion (log_2_ >3 or < −3, *p* < 0.015) was applied, we found that 14,654 genes were differentially expressed in the AZ relative to the NAZ or changed more than eight-fold in LAZ over time (Figure 1E and Table S1). To be consistent, we have included both higher (log_2_ > 3) and lower (log_2_ < −3) LAZ/NAZ gene expression; nonetheless, we recognize that there is a difference between the absence of a TF in the AZ, which might be a repressor of gene expression, and the absence of an enzyme or structural protein in the AZ. There were 13,969 genes whose expression changed more than eight-fold at any time collection between the 0 and 72 h treatment and 1,845 genes whose expression was abscission-specific (higher or lower in the LAZ relative to the NAZ; Figure 1E). Within this grouping were 1,160 genes whose expression was both abscission-specific and changed more than eight-fold over time (Table S1). These 1,160 DEGs may be regulated or co-regulated by the 118 TFs that were similarly expressed. Below we focus our analysis on the 188 abscission-specific TFs and the 1,845 potential targets for these TFs that we predict are specifically involved in the abscission process. We have included in our analysis the 70 TFs (Figure 1D) and 685 DEGs (Figure 1E) that were abscission-specific but did not change more than eight-fold over time because by simply being abscission-specific may indicate a special role in abscission.

### Characterization of abscission-specific transcription factors in soybean

**Figure 2:**
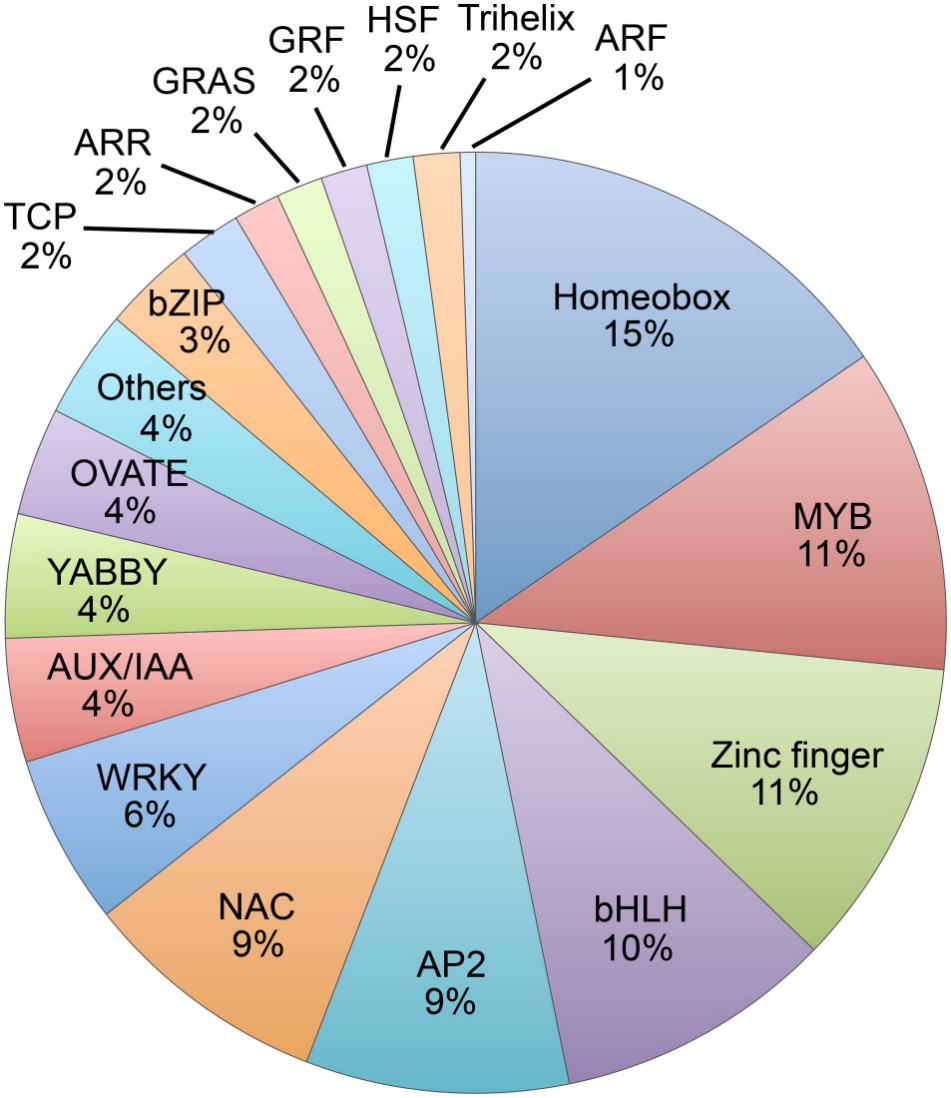
**Pie chart of families of abscission-specific transcription factors differentially expressed in soybean leaf abscission**. The chart displays the gene family classification of the 188 abscission-specific TFs that were differentially regulated more than eight-fold in the LAZ relative to NAZ (transcripts in LAZ/NAZ at 0, 12, 24, 48, or 72 h, and 118 that also changed more than eight-fold over time and LAZ at 12/0, 24/0, 48/0, or 72/0 h).

Among the 188 abscission-specific TFs, there were 18 different families of TFs including homeobox, MYB, various types of Zinc finger, bHLH, AP2, NAC, WRKY, YABBY (YAB), IAA, and others (Figure 2 and Table S2), suggesting a complex regulation of organ separation. In particular, over 15% (29 of 188) of the abscission-specific TFs contained a homeobox domain and this was followed by MYB (21 of 188, 11%), Zinc finger (20 of 188, 11%), bHLH (18 of 188, 10%), and AP2 domain TFs (17 of 188, 9 %). In addition, there were several hormone related TFs represented by AUX/IAA (8 of 188, 4%), ARR (3 of 188, 2%), and ARF (1 of 188, 1%). Of particular interest in this group was the plant-specific YABBY family of TFs, which accounted for more than 4% (8 of 188) of the TFs. The YABBY gene family is a relatively small family of genes found only in the plant kingdom. Moreover, YABBY gene expression was most abundant in the group with higher expression in the LAZ and not highly expressed in the NAZ (Table 1, Table S2).

**Figure 3:**
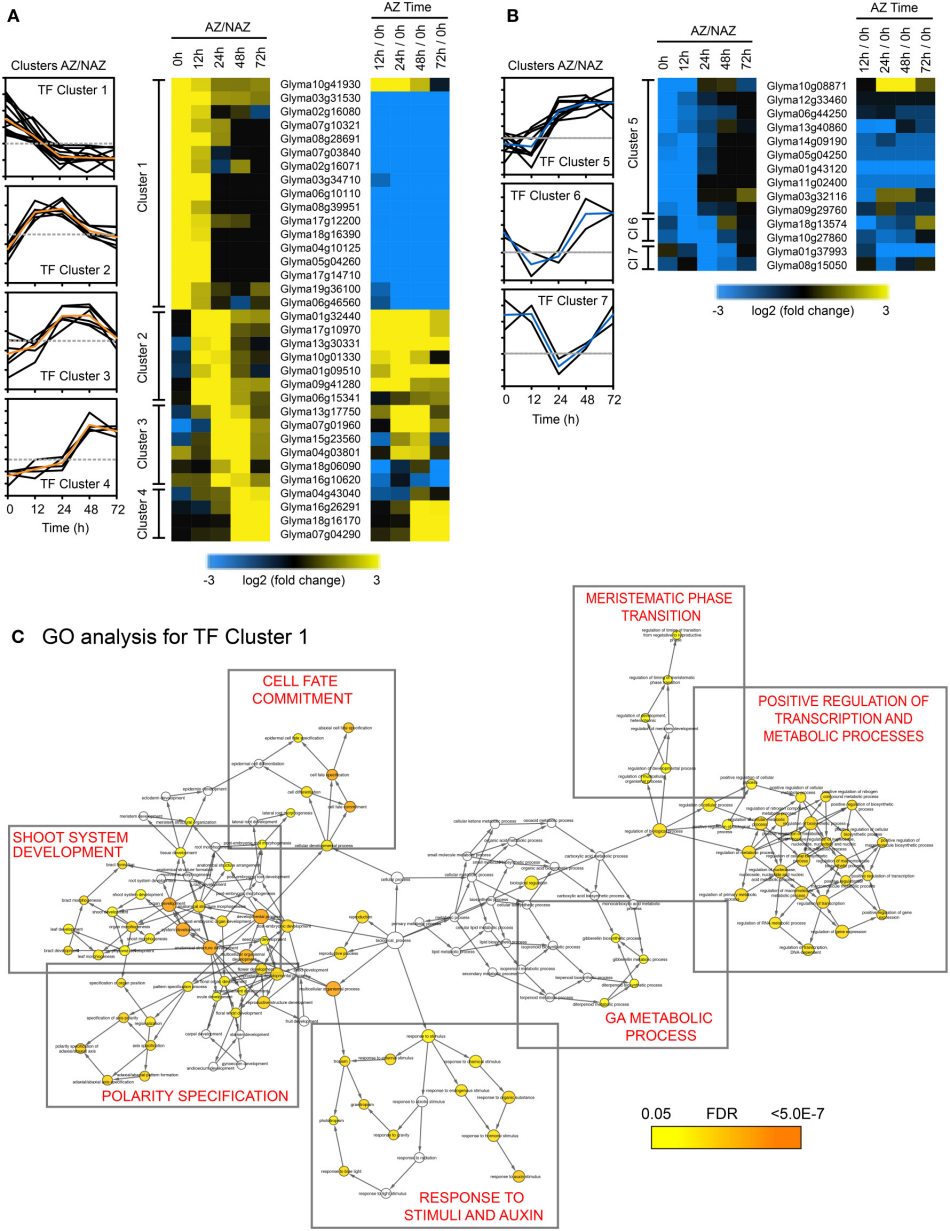
**Cluster analysis of abscission-specific transcription factors (188 TFs) more or less abundant in the AZ at two consecutive time points in soybean leaf abscission (48 TFs). (A)** Heat map display of the 34 abscission-specific TFs that clustered based on expression greater than four-fold in the LAZ relative to the petiole (NAZ) (log_2_ > 2, *p* < 0.015) in two consecutive time points (i.e., LAZ/NAZ at 0 and 12 h, 12 and 24 h, 24 and 48 h, and 48 and 72 h). Change in expression for the same TFs in the LAZ over time (i.e., expression in LAZ at 12/0, 24/0, 48/0, and 72/0 h). **(B)** Similar heat map display of the 14 abscission-specific TFs that clustered based on expression of four-fold less in the LAZ relative to the NAZ (log_2_ < −2, *p* < 0.015) in two consecutive time points. **(C)** Gene Ontology (GO) term network analysis (BiNGO) for TF Cluster 1 having four-fold higher expression in the LAZ/NAZ at 0 and 12 h. Enrichment clusters with similar biological processes are boxed and a summary of the biological process is printed in red inside the box. The range of colors from yellow to orange inside the circles for each identified biological process indicates the statistical significance from 0.05 to < 5 × 10^−7^, respectively, for the enrichment of the GO term in the test set, Cluster 1 TFs, (Maere et al., 2005). The color bar at the bottom right reflects the range of statistical significance where the *p*-value was adjusted using a Benjamini and Hochberg False Discovery Rate (FDR) correction.

As mentioned above, a working model for abscission predicts four developmental phases that culminate in organ separation and synthesis of a protective scar: Phase (1) establishment of the AZ; Phase (2) acquisition of competence to respond to abscission signals; Phase (3) activation of abscission/cell separation; and Phase (4) trans-differentiation between the separating sides of the AZ cells and deposition of a protective layer (Bleecker and Patterson, 1997; Lewis et al., 2006; Liljegren, 2012; Kim, 2014; Tucker and Kim, 2015). Because abscission is a nexus of multiple developmental and environmental signals, abscission should be thought of as a mutually interconnected process that occurs within a developmental time frame (Tucker and Kim, 2015). To identify transcriptional regulation that underlies the developmental processes coordinating organ separation, we performed a cluster analysis of the 188 abscission-specific TFs to group TFs that presumably share a common role in the regulation of the different phases of soybean leaf abscission. The 188 TFs were clustered based on a minimum expression of >four-fold up- or down-regulation in two consecutive time points (log_2_ > 2 or < −2, *p* < 0.015; i.e., LAZ/NAZ at 0 and 12 h, 12 and 24 h, 24 and 48 h, 48 and 72 h; Figures 3A,B). We chose to assess a sustained differential expression over two consecutive time points and not just one or more than two so that we could be more certain that the differential expression of a TF could be linked to a particular process or phase of abscission. A total of seven clusters were identified, which included 48 of the original 188 genes (Figure 3 and Tables S2, S3). Four clusters included genes that were more highly expressed in the AZ than the petiole (34 genes) and three clusters where the genes were more highly expressed in the petiole (14 genes). TF Cluster 1, which included genes that were higher in the LAZ than NAZ (log_2_ > 2) at 0 and 12 h, contained the greatest numbers of TFs (17 genes; Figure 3A). All but 1 of the genes in Cluster 1 declined with time. Because organ separation at the LAZ was not detected until 48 h after ethylene treatment began, we presume that the Cluster 1 TFs are associated primarily with phase 2 of abscission, i.e., acquisition of competence to respond to abscission signals. Gene Ontology (GO) analysis of TF Cluster 1 was enriched for biological processes associated with shoot system development, polarity specification of adaxial/abaxial axis, cell fate commitment/specification, reproductive structure development, response to endogenous/external stimuli, response to auxin, gibberellin metabolic process, regulation of the timing of meristematic phase transition, and positive regulation of transcription and metabolic processes (Figure 3C).

Of special interest in TF Cluster 1 were TFs encoding plant specific YABBY family proteins, which included INNER NO OUTER (INO), ABNORMAL FLORAL ORGANS/FILAMENTOUS FLOWER (AFO/FIL), YAB2, and YAB5. Current annotation for the YABBY TF family proteins predicts six members in Arabidopsis (Siegfried et al., 1999; Plant TF Database http://plntfdb.bio.uni-potsdam.de/v3.0/) and 17 members in soybean (PlantTFDB at http://planttfdb.cbi.pku.edu.cn). There are five subfamilies of YABBY in angiosperms represented by AFO/YAB3, YAB2, YAB5, CRAB CLAW (CRC), and INO clades (Bonaccorso et al., 2012; de Almeida et al., 2014). It is therefore noteworthy that 6 out of the 17 TFs in TF Cluster 1 were YABs, which declined rapidly after induction of abscission (Tables S2, S3).

Also included in TF Cluster 1, were soybean TFs orthologous to HOMEOBOX 1 (ATHB-1) and LATE MERISTEM-IDENTITY-1 (LMI1/HOMEOBOX 51/ATHB-51). Both ATHB-1 and LMI1 have been demonstrated to affect cell fate and development (Aoyama et al., 1995; Saddic et al., 2006). Overexpression of ATHB-1 in tobacco resulted in de-etiolated seedlings in the dark and leaves in the light with light green sectors of spongy parenchyma cells rather than dark green palisade cells (Aoyama et al., 1995). It was concluded that ATHB-1 was a positive transcriptional activator that altered cell fate (Aoyama et al., 1995). LMI1 (ATHB-51) is a positive regulator of genes that regulate organ identity in the meristem (Saddic et al., 2006; Grandi et al., 2012).

Although TF Cluster 2 (high in both 12 and 24 h) did not predict any enriched biological processes, this cluster contained transcriptional co-regulators most similar to Arabidopsis OVATE 2 and OVATE 4. Also in TF Cluster 2 is a NAC domain protein that was both higher in the LAZ than NAZ and strongly up-regulated over time. Also in Cluster 2 was an AP2/ERF TF, which suggests that an ethylene response had begun in the AZ by 12 h. TF Cluster 3, which spans the 24 and 48 h time points, is enriched in GO terms for response to endogenous and hormone stimulus, ethylene-mediated hormone signaling (Figure S1A), and reproductive development, which included ETHYLENE AND SALT INDUCIBLE 3 (ESE3), GATA 9 and ABERRANT TESTA SHAPE/KANADI 4 (ATS/KAN4) (Tables S2, S3). In our explant system, ~10% of the upper AZ had separated by 48 h (Figure 1C). Presumably, changes in TFs associated with phase 3 of abscission, activation of abscission, began at 12 h when an increase in the ETHYLENE RESPONSE FACTOR (ERF) was observed and continued at least until 48 h when organ separation was detected. Thus, gene expression of TFs in TF Clusters 2 and 3 are most likely linked to activation of abscission.

TF Cluster 4, which spans the 48 and 72 h time collections, and most likely associated with phase 4 of abscission, trans-differentiation of proximal and distal cells, includes WRKY 72, DEHYDRATION RESPONSE ELEMENT-BINDING PROTEIN 26 (DREB26), INDUCER OF CBF EXPRESSION 1 (ICE1). GO term analysis of this cluster indicates that these TFs are best linked to responses to stress and, curiously, stomatal development (Figure S1B).

The second largest grouping in our TF cluster analysis is TF Cluster 5, which included 10 genes whose expression was lower in the AZ and higher in the petiole at 0 and 12 h. As we concluded for TF Cluster 1 genes, we presume that at least some of the genes in TF Cluster 5 are linked to phase 2 of abscission, acquisition of competence to respond to abscission signals. Because gene expression for these TFs is higher in the petiole than the LAZ, if these TFs are important to abscission, their absence from the LAZ would make possible the activation or binding of other TFs that evoke a differential gene expression linked to phase 3, activation of abscission. TF Cluster 5 was enriched in GO terms for asymmetric division and multicellular organismal development (Figure S1C and Tables S2, S3). The GO terms associated with TF Cluster 1 and 5 suggest that these 27 genes play critical roles in the control of cell fate and response to plant hormones, which includes auxin, cytokinin, gibberellic acid (GA), and ethylene. As expected, because the source of auxin was removed, TFs associated with auxin signaling (AUX/IAA) or co-regulators of auxin were rapidly down-regulated over time (Table S2).

Also in TF Cluster 5 were five NAC domain containing TFs. These NAC TFs, which were less abundant in the LAZ than the petiole are in contrast to the strongly AZ-specific NAC TF in Cluster 2 (Tables S2, S3). Moreover, there were several more NAC domain TFs that were either more abundant in the LAZ than the petiole or the reverse but did not fall into our clustering restriction of only two consecutive times (Table S2). NAC TFs are one of the largest families of TFs in the plant kingdom that regulate a variety of developmental and environmental responses (Olsen et al., 2005). Interestingly in regard to NAC domain TFs, when an 18 bp element (Z-BAC) from the promoter of the *BEAN ABSCISSION CELLULASE 1* (*BAC1*) (Tucker et al., 2002) was used in a yeast one-hybrid screen to identify putative DNA-binding proteins in a bean (*Phaseolus vulgaris*) leaf abscission zone (LAZ) cDNA library, the most common clones identified were for a NAC domain protein (*PvNAC1*) (results not shown). When the *PvNAC1* TF was co-expressed with a BAC1::luciferase promoter construct in a transient bean expression assay (Tucker et al., 2002), expression of the BAC promoter was suppressed by 84%. However, we were unable to demonstrate direct binding of the NAC protein to the *BAC1* promoter using an electrophoretic mobility shift assay (results not shown). Nonetheless, the dissimilar expression patterns of the NAC TFs accentuate the potential for both positive and negative regulation of gene expression by the same family of TFs (Olsen et al., 2005; Jin et al., 2015).

### Clustering of differentially expressed genes (DEGs) in the entire transcriptome

**Figure 4:**
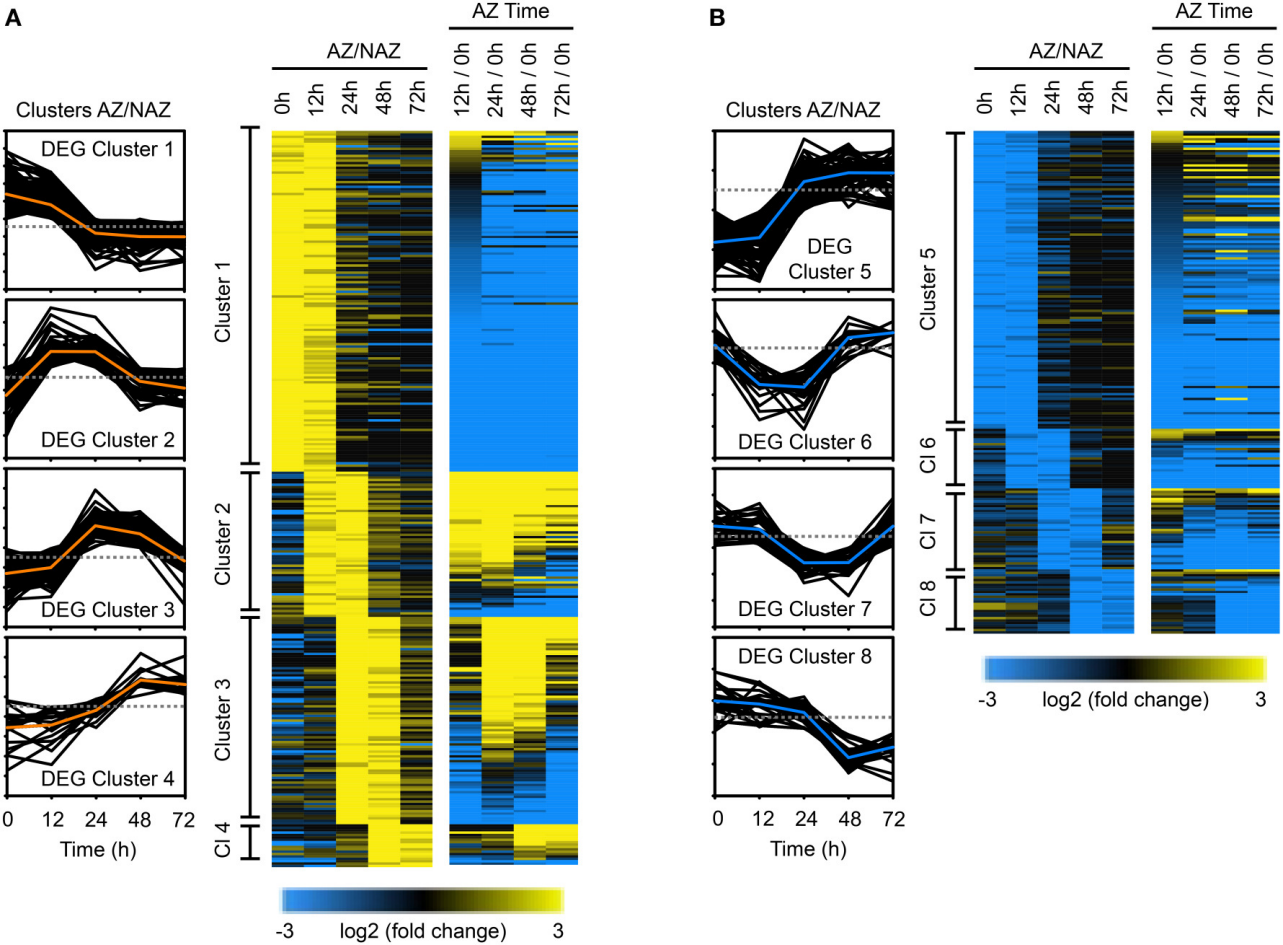
**Cluster analysis of differentially expressed genes (1,845 DEGs) in the entire transcriptome in two consecutive time points during soybean leaf abscission (520 DEGs)**. **(A)** Heat map display of the 309 abscission-specific differentially expressed genes (DEGs) in entire transcriptome that clustered based on expression greater than four-fold in the LAZ/NAZ (log_2_ > 2, *p* < 0.015) in two consecutive time points and change in expression over time (e.g., 12/0 h, see Figure 3A). **(B)** Similar heat map display of the 211 abscission-specific DEGs that clustered based on four-fold less expression in the LAZ/NAZ (log_2_ < −2, *p* < 0.015) in two consecutive time points and change in expression over time (see Figure 3B).

To gain insights into potential downstream events associated with the 188 abscission-specific TFs identified above, we examined changes in the soybean transcriptome having a similar pattern of up or down regulation in two consecutive times. When the same cutoff for expression levels (log_2_ > 2 or < −2, *p* < 0.015) was applied to cluster the abscission-specific genes (1,845 genes), we identified eight clusters that spanned two consecutive time points in an abscission-specific manner (Figure 4). The first four clusters were comprised of genes that were more abundantly expressed in the AZ (Figure 4A) and the latter four clusters contained genes that had lower abundance in the AZ relative to the petiole (Figure 4B). As was observed for the TF clusters, the first cluster (higher in the AZ than NAZ at 0 and 12 h) and the fifth cluster (lower in the AZ than NAZ at 0 and 12 h) contained the greatest numbers of genes. DEG Cluster 1 included 143 genes (Figure 4A and Table S4). GO term analysis of DEG Cluster 1 predicted similar but distinct results compared to that of TF Cluster 1 (Figure S2A). In addition to biological processes associated with response to endogenous/external stimuli, response to auxin, and reproductive structure development, the GO analysis of DEG Cluster 1 included cellular response to phosphate starvation, cytokinin transport, cutin transport, myo-insitol transport, flavonoid biosynthetic processes, cellular amino acid derivative, and metabolic processes. GO analysis of DEG Cluster 1 indicates that gene expression at 0 and 12 h is associated with inter-cellular communication and metabolic processes associated with an early abscission response. Moreover, GO analysis of DEG Cluster 1 suggests that the AZ cells have begun to respond to changes in cellular nutrition, which may be linked to the removal of the leaf blade. Also, as expected, expression of SAUR genes (SMALL AUXIN UPREGULATED RNA) declined rapidly from 0 to 12 h (Figure 4A and Table S4).

DEG Cluster 2 (61 genes), spanning the 12 and 24 h collections (Figure 4A and Table S4) includes homologs of ACC SYNTHASE 10, INFLORESCENCE DEFICIENT IN ABSCISSION (IDA)-like 1 and basic chitinase (PR-3), which have been previously linked to early stages of abscission in several species (Bleecker and Patterson, 1997; Roberts et al., 2002; Butenko et al., 2003; Meir et al., 2010; Tucker and Yang, 2012). GO term analysis of DEG Cluster 2 confirmed the abscission-specificity of this cluster (Figure S3A). DEG Cluster 3 at 24 and 48 h included 87 soybean genes enriched in GO terms for carbohydrate metabolic process and regulation of cell size (Figure S3B). Many of the genes in this cluster are annotated as cell wall hydrolytic and cell wall modifying enzymes that reflect the actual cell separation process (Figure 4A and Table S4). Although 18 genes grouped into DEG Cluster 4 spanning 48 and 72 h, there were no GO term predictions associated with these genes; nonetheless, this cluster included an arabinogalactan protein, which may reflect changes in the cell wall structure.

DEG Cluster 5, the second largest DEG cluster, included 126 genes that were more highly expressed in the petiole than the LAZ (Figure 4B and Table S4). GO term analysis of DEG Cluster 5 highlighted processes associated with cellulose biosynthesis, secondary cell wall biogenesis, rhamnogalacturonan I side chain metabolism, and proteolysis (Figure S2B). In addition, DEG Cluster 5 was also associated with biological processes for positive regulation of abscisic acid (ABA) and lipid biosynthesis (Figure S2B). It should be noted here that to be consistent with the clustering of TFs, we included DEGs that were more abundant in the petiole (higher in the NAZ than LAZ); however, as noted above, the lack of a TF in the AZ, which might be a repressor, is different than the lack of an enzyme in the AZ. A higher concentration of a DEG in the petiole at 0 and 12 h may reflect differences in processes that were occurring in the petiole at the time of harvest and may not be directly linked to abscission, e.g., cellulose biosynthesis.

GO term analysis for DEG Cluster 6 and 7 (less abundant in the LAZ at 12 and 24 h, and 24 and 48 h, respectively) did not highlight any special process; however analysis of DEG Cluster 8 (48 and 72 h) highlighted gene expression associated with response to stress and heat (Figure S3C), which might reflect the induction of programmed cell death and senescence at this time interval.

### Regulatory networks of transcription factors in soybean leaf abscission

Although genomic-scale studies using microarray and RNA-seq have provided considerable data on TF gene expression in a variety of plant tissues in many plant species, information on the regulatory networks governed by TFs in species other than Arabidopsis is still lacking (Rhee and Mutwil, 2014). It is generally accepted that almost all eukaryotic genes are regulated by more than one TF and their target genes are also dependent on several TFs and/or co-regulators (Sorrells and Johnson, 2015), and a study of the transcriptional networks in Arabidopsis confirmed the importance of regulatory pairs of TFs (Jin et al., 2015). Moreover, the interactions of TFs in a regulatory network appear to be conserved during the evolution of multicellular organisms (Jin et al., 2015) in highly diverse species (Sorrells and Johnson, 2015). An interesting finding in the Arabidopsis study was that regulatory networks associated with development had more TFs and co-regulators per target gene than environmental or stress response networks (Jin et al., 2015). This would suggest that gene expression associated with developmental responses is more finely and discretely regulated than environmental responses. Thus, abscission, which integrates both environmental and developmental cues, may be a complex interplay of several regulatory networks.

**Figure 5:**
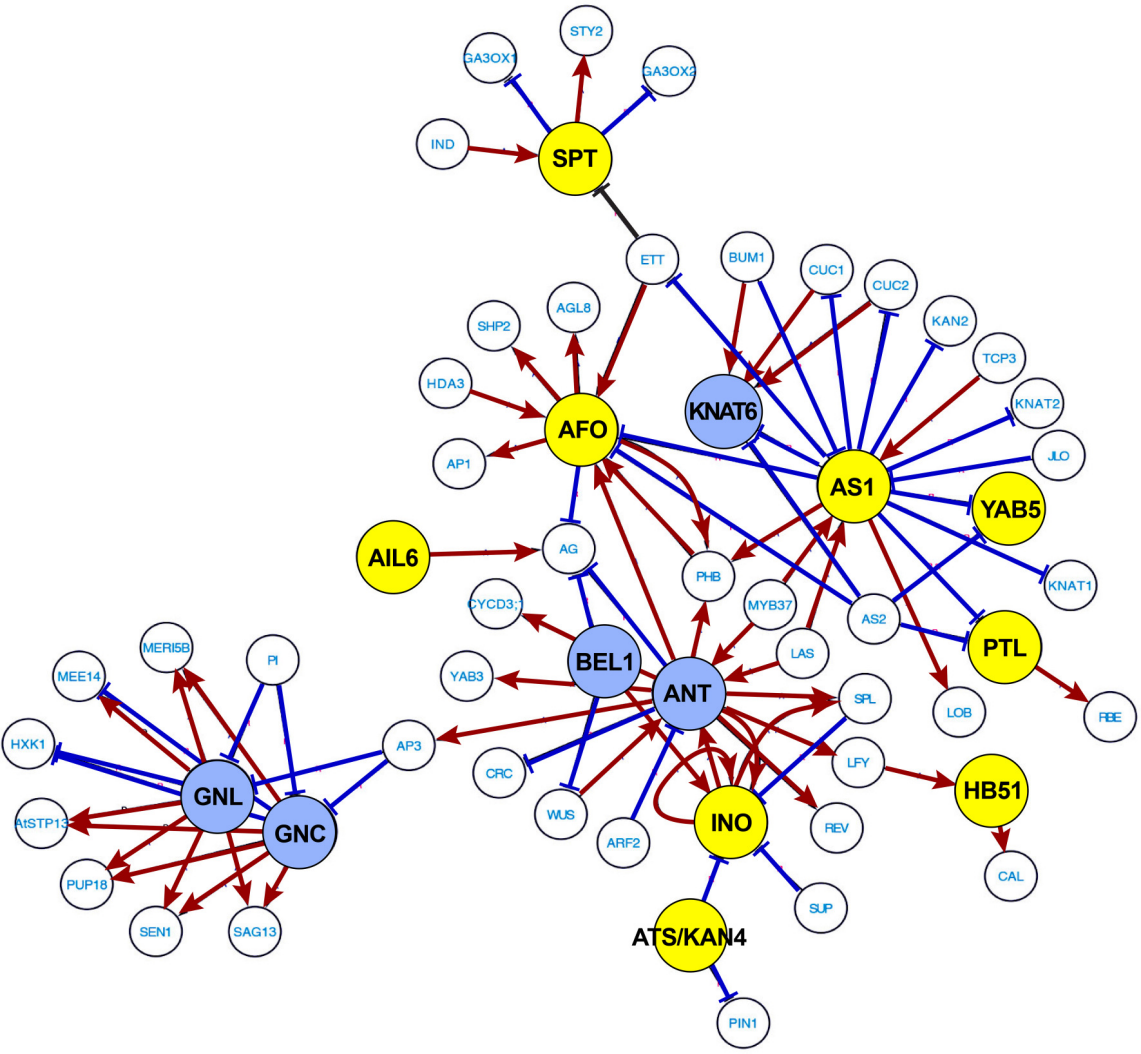
**Most extensive transcriptional network underlying soybean leaf abscission**. The 188 abscission-specific soybean TFs corresponding to 133 Arabidopsis TFs (Table S2) were used to construct high-confidence transcriptional networks using the Arabidopsis Transcriptional Regulatory Map (ATRM). Of 133 Arabidopsis TF homologs, only 40 of Arabidopsis TFs were found in the ATRM data set (Table 1, Table S5). Arabidopsis TF orthologs identified in expression data of soybean leaf abscission were color-coded. Soybean TFs eight-fold higher in the LAZ/NAZ are highlighted in yellow and TFs eight-fold lower in the LAZ/NAZ are highlighted in blue. Visualization of the network was generated by Cytoscape v. 3.2.1. Red arrows indicated a positive regulation and blue bars a negative regulation of the target TFs or co-regulators generated from ATRM data set.

Above we focused on GO term analysis to identify developmental and environmental processes linked to abscission. To further substantiate and investigate transcriptional regulation of soybean leaf abscission, we used the publicly available data from the Arabidopsis Transcriptional Regulatory Map (ATRM) (Jin et al., 2015). In the 188 soybean abscission-specific TFs discussed above (Figure 1D), there were 133 different Arabidopsis TFs represented (Table S2). Among the 133 TFs, 40 TFs were found in the high-confidence regulatory ATRM data set (Table 1). These 40 were used to predict several interactive transcriptional networks for soybean leaf abscission (Figure 5 and Figure S4). The largest network (Figure 5), which is discussed in the sub-sections below, consisted of TFs with domains for: AP2 [e.g., AINTEGUMENTA (ANT) and AINTEGUMENTA-like 6 (AIL6)], ASYMMETRIC LEAVES 1 (AS1), homeobox [e.g., KNOTTED-like 6 (KNAT6), ATS/KAN4, LMI1/HOMEOBOX 51, and BEL1], YABBY (e.g., INO, AFO, and YAB5), Zinc finger [e.g., GATA, NITRATE-INDUCIBLE, CARBON-METABOLISM INVOVLED (GNC) and CYTOKININ-INDUCED GATA1/GNC-like (GNL)], and Trihelix [e.g. PETAL LOSS (PTL)].

#### TFs associated with stem cell maintenance

ANT and AIL6 encode Arabidopsis TFs that belong to an eight-member subfamily of AIL/PLETHORA (PLT) in the larger AP2/ERF TF family (Nole-Wilson et al., 2005; Prasad et al., 2011). In Arabidopsis, it was demonstrated that ANT and AIL6 are associated with various developmental processes including the maintenance of shoot and flower meristems, organ size, flower initiation, and floral organ identity. Mutational studies of the *AIL* genes support their regulatory role in meristems as shown by loss or reduced growth of organs (loss-of-function) and ectopic formation or increased growth of organs (gain-of-function; Krizek, 2009; Horstman et al., 2014). The defects of *ant* and *ail6* mutants correlate with expression of the stem cell and floral organ regulatory genes, and changes in the expression of auxin responsive and transport genes (Krizek, 2009). Based on expression of the *ANT* gene in the *arf2* mutant, it was suggested that *ANT* might be regulated by ARF2 (Schruff et al., 2006). The results collectively suggest that ANT and other AIL TFs control the balance between cell proliferation and differentiation in response to auxin gradients that maintain growth and patterning in different developmental processes (Krizek, 2009).

The expression patterns of the soybean *ANT* and *AIL6* genes suggest a complex role in leaf abscission. Expression of *ANT* is higher in the petiole than the LAZ at 0, 12, and 24 h, but *AIL6* is significantly higher in the LAZ at 48 h than the petiole (Figure 5 and Table 1), indicating that soybean AIL TFs may have distinct roles in leaf abscission. In fact, genetic studies in Arabidopsis and expression studies in other species demonstrated that AIL TFs have distinct and differing roles depending on the systems studied (Rieu et al., 2005; Mudunkothge and Krizek, 2012; Horstman et al., 2014). In regard to *ANT* and *AIL6* expression in response to auxin, it is worth noting that the ARF7/19 and ARF1/2 are involved in the regulation of floral organ abscission in Arabidopsis (Ellis et al., 2005). Working forward from the Arabidopsis results, it would be interesting to know if an auxin-ANT/AIL module regulates the balance between cell proliferation and differentiation through translating an auxin gradient that might form during soybean leaf abscission (Louie and Addicott, 1970; Tucker and Kim, 2015).

#### TFs associated with hormone signaling pathways

In the transcription network shown in Figure 5, ANT and AIL6 are associated with AP3, which is a floral organ regulatory gene in Arabidopsis that is connected to the regulation of GNC-GNL. GNC and GNL were originally identified in gene expression studies with nitrate, cytokinin, and light treatments of Arabidopsis (Bi et al., 2005; Naito et al., 2007). Subsequent studies demonstrated that auxin and GA also regulate *GNC* and *GNL* expression. GNC and GNL act as negative regulators of germination, GA catabolism (GA20X2), cotyledon expansion, flowering time, senescence, and floral organ abscission (Richter et al., 2013; Behringer and Schwechheimer, 2015). Of particular interest is that over-expression of GNC and GNL share many phenotypes with *arf2* mutants (Behringer and Schwechheimer, 2015), which as mentioned above display a delay in floral organ abscission (Ellis et al., 2005; Okushima et al., 2005; Schruff et al., 2006). Moreover, ARF2 and ARF7 were demonstrated to directly bind to the promoters of *GNC* and *GNL* (Richter et al., 2013). These observations further support the putative role of auxin- and GA-mediation of *GNC* and *GNL* expression in the control of abscission.

In our soybean RNA-seq data, the expression of *GNC* and *GNL* homologs are more strongly expressed in the petiole than the LAZ (Figure 5 and Table 1) and their lower expression or lack of expression in the LAZ may be important to soybean leaf abscission. Moreover, considering that ANT and AIL6 proteins, like GNC and GNL, are downstream components of the ARF2/ARF7-mediated signaling module, it is possible that the GNC-GNL and ANT-AIL6 pair of co-transcriptional regulators may play an important role in the transcriptional regulation of organ abscission in plants. This is consistent with their role described above to control the balance between plant hormone signaling pathways in plant growth and development.

#### TFs associated with organ polarity and growth

The transcriptional network analysis indicates that ANT-AIL6 and GNC-GNL are associated with meristem TFs affecting organ polarity and organ boundary determinants, which includes YAB-ATS/KAN4, AS1, and KNAT6 (Figure 5). The *YABBY* gene family was briefly discussed above because members in this family were prominent in TF Cluster 1. Three members of the *YABBY* gene family (*AFO, INO*, and *YAB5*) were also prominent in the transcriptional regulatory network generated with the ATRM data (Jin et al., 2015; Figure 5). In Arabidopsis, YABs regulate growth of the integuments and lateral organs, which includes leaves, sepals, petals, and carpels (Eshed et al., 1999, 2001, 2004; Kerstetter et al., 2001). In particular, the *INO* gene is expressed in the outer integument of the ovule (Villanueva et al., 1999) and the *AFO/FIL, YAB3, YAB2*, and *YAB5* genes are expressed in the abaxial domain of developing leaf and floral organ primordia (Siegfried et al., 1999; Sarojam et al., 2010). It was concluded that YAB gene expression is critical to organ polarity and subsequent lamina growth and cell identity in developing organs (Eshed et al., 1999, 2004; Sawa et al., 1999; Siegfried et al., 1999; Stahle et al., 2009; Sarojam et al., 2010). YABs (i.e., AFO/FIL, YAB3, YAB2, YAB5) repress expression of shoot apical meristem (SAM) regulatory genes in the developing leaf primordia (Bonaccorso et al., 2012). Without YAB activity, expression of *KNOX* and *WUS* genes were up-regulated in leaves, which caused the formation of meristem-like structures on the leaves (Kumaran et al., 2002; Sarojam et al., 2010). The soybean *INO, AFO*/*FIL, YAB2*, and *YAB5* genes are highly expressed in the LAZ at 0 and 12 h (Figure 5, Table 1; Tables S2, S3). In contrast, expression of a soybean *KNAT6* gene is repressed prior to and at the beginning of abscission at 0, 12, and 24 h (Figure 5 and Table 1). Their expression pattern suggests that YAB-KNAT6 may contribute to defining the organ separation boundary by suppressing the AZ cell proliferation or promoting de-differentiation of cells at the onset of abscission similar to their role in the meristem.

Also in the ATRM generated TF network is a KAN TF (ATS/KAN4) that can interact with the YAB protein (INO). Arabidopsis gene expression and mutant analysis of *ATS*/*KAN4* indicate that it plays a role in defining the organ boundary that separates the two integuments (inner and outer integuments; McAbee et al., 2006). It was concluded that *ATS*/*KAN4* gene expression regulates organ polarity and boundary formation necessary for proper integument growth as it does for abaxial identity during leaf development. Increased expression of soybean *ATS*/*KAN4* genes (Figure 5 and Table 1) during organ separation at 24 and 48 h may indicate that the soybean *ATS*/*KAN4* gene may be involved in defining the separation boundary of the AZ. Studies of *ATS*/*KAN4* and *YABs* in Arabidopsis and their expression patterns in soybean leaf abscission collectively emphasizes the importance of a balance among diverse polarity determinants in the control of abscission.

Other boundary determinants critical to organ polarity and cell fate identified in our study include a soybean homolog of *AS1* (Figure 5 and Table 1). In Arabidopsis, *AS1* is also required for organ boundary/polarity, cell fate, and proper establishment of floral organ AZ cells (Byrne et al., 2000; Hazen et al., 2005; Gubert et al., 2014). It was suggested that Arabidopsis AS1 controls the placement of the sepal and petal abscission zones, which appear to affect either the timing of abscission zone differentiation or the activation of cell separation (Gubert et al., 2014). The soybean *AS1* gene is most highly expressed in the LAZ at 12 h before or at the beginning of actual cell separation (Figure 5 and Table 1).

In Arabidopsis, AS1, together with AS2, repress expression of *KNOTTED1-LIKE HOMEODOMAIN* (*KNOX*) genes, which are meristem-promoting genes affecting leaf development (Ori et al., 2000; Guo et al., 2008; Xu et al., 2008). Moreover, AS1 acts upstream of BREVIPEDICELLUS (BP, KNAT1), KNAT2, and KNAT6 homeodomain TFs that have been demonstrated to affect development of the floral organ AZ in Arabidopsis. In Arabidopsis, *BP/KNAT1* represses expression of *KNAT6* (Shi et al., 2011). Although *KNAT1* expression is not restricted to the AZ in Arabidopsis, *KNAT6* expression was higher in the AZ compared to surrounding tissues (NAZ; Shi et al., 2011). In soybean, expression of *KNAT6* (*KNOTTED1-LIKE HOMEODOMAIN 6*) was lower in the AZ compared to the petiole at 0, 12, and 24 h (Table 1). It appears that abscission-specificity of *KNAT6* in soybean is different than in Arabidopsis; nonetheless, the interactions of soybean *AS1, KNAT1*, and *KNAT6* in the development of the AZ might still be of interest because *AS1* and *KNAT6* gene expression was significantly different between the LAZ and petiole and this may influence development of the AZ or formation of the separation layer in soybean.

Also of potential interest in regard to defining the separation layer within the soybean LAZ are TFs for soybean BELL-type homeodomain and PETAL LOSS (PTL). In Arabidopsis, the BELL-type homeodomain TF was shown to repress growth in the boundary region between the floral organ and flower receptacle, and also be required for establishment of stamen AZ (Gómez-Mena and Sablowski, 2008). In addition, PTL was suggested to act as a negative regulator of cell proliferation in the floral organs of Arabidopsis. Expression of the soybean *BEL1* gene is lower in the LAZ than the petiole and in contrast the *PTL* genes were more highly expressed in the LAZ at 0 and 12 h and declined over time (Figure 5 and Table 1; Table S2). Gene expression profiles for these TFs support their potential antagonistic role in restricting cell proliferation and organ boundary determination in the soybean AZ.

## Concluding remarks

**Figure 6:**
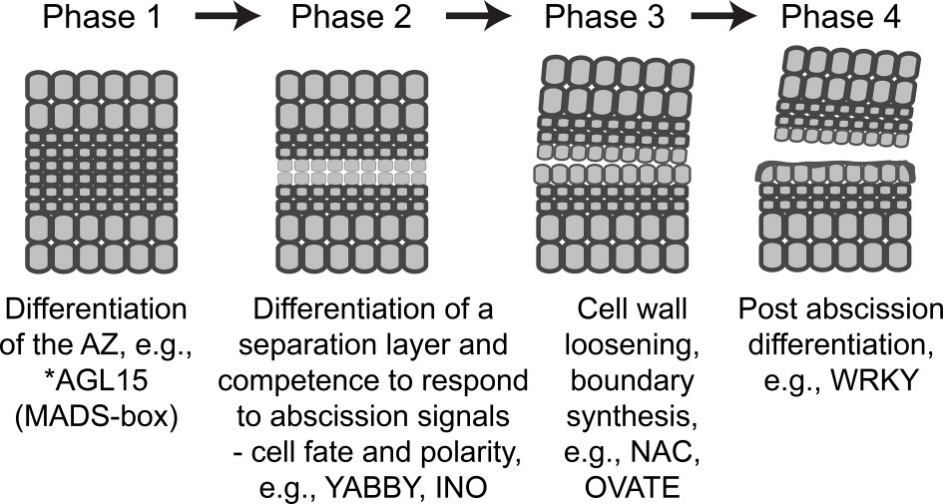
**Revised abscission model depicting each of the four phases of abscission and the most prominent TFs associated with each phase**. The descriptions for phases 2 and 3 have been revised to reflect the findings in our study. ^*^ No soybean *AGL15*-like gene was expressed in our RNA-seq results; however, others have demonstrated its importance to formation of the Arabidopsis floral AZ and we have placed it into phase 1 (Fernandez et al., 2000) similar to the placement of the tomato *JOINTLESS, MADS-box* gene (Mao et al., 2000; Nakano et al., 2012). The up and down-regulated expression patterns for *YABBY, INO, NAC, OVATE*, and *WRKY* genes can be found in Table S2.

In the current study, we identified 188 soybean TFs that were differentially regulated more than eight-fold in an abscission-specific manner. Cluster analysis of these abscission-specific TFs requiring an up or down-regulation in two consecutive time points enabled us to group soybean TFs that may share similar functions in the progression of organ abscission and development of the AZ cells. Of particular interest was TFs that were more highly expressed in the AZ relative to the petiole at 0 and 12 h (TF Cluster 1). Many of the TFs identified within this cluster are known as key determinants in maintenance of organ polarity, lateral organ growth and cell fate. The association of these TFs with organ polarity and boundary definition was further substantiated in the transcriptional networks generated using the ATRM data set (Jin et al., 2015), which suggests that these TFs may play a role in defining the separation layer within the multilayer LAZ. The most prominent TFs associated with each of the phases of abscission are listed in the abscission model depicted in Figure 6. We propose that the TFs listed in the model function as transcriptional regulators to balance plant hormone signaling, organ polarity, and meristem-like responses in the AZ cells prior to the onset of organ separation.

Identification of meristem-associated genes in the AZ has been noted by others (Nakano et al., 2013; Wang et al., 2013). In addition, a recent publication further substantiates the concept that meristem-associated genes that control formation of specialized domains of restricted growth known as boundaries between stem cell activity of shoot apical meristem and lateral organ growth may also underlie boundaries formed during organ abscission (Hepworth and Pautot, 2015). It seems logical that organ (leaf, flower, fruit, etc.) abscission would be an adaptation of a primal process in the meristem. Processes and signals that regulate organ polarity and boundary formation in the meristem might therefore be similarly found in the AZ. Nonetheless, the functional relevance of these TFs in abscission awaits further experimentation. Identification of the transcriptional targets for these TFs will provide a better understanding of how these conserved gene networks control plant development. In regard to abscission, most plant AZs are comprised of several layers of small, less-vacuolated cells (Roberts et al., 2002). The signals and factors that determine where within this AZ the separation layer will form are not known (Tucker and Kim, 2015). It will be interesting to determine in the future if some of the TFs identified in this study are involved in establishing the position of the separation layer within the AZ.

## Author contributions

JK and MT, plant and RNA sample preparation, data analysis and manuscript preparation; RY, plant and RNA sample preparation; JY, RS, and CC, data analysis and manuscript preparation.

## Funding

This work was supported by a Binational Agricultural and Development Fund (BARD) US-4571-12C grant to MLT.

### Conflict of interest statement

The authors declare that the research was conducted in the absence of any commercial or financial relationships that could be construed as a potential conflict of interest.

## Supplementary material

Figure S1 **GO term analysis of abscission-specific TF Clusters 3, 4, and 5 (Figures 3A,B)**.

Figure S2 **GO term analysis of abscission-specific DEG Clusters 1 and 5 for the entire transcriptome (Figures 4A,B)**.

Figure S3 **GO term analysis of abscission-specific DEG Clusters 2, 3 and 8 for the entire transcriptome (Figures 4A,B)**.

Figure S4 **Less extensive transcriptional networks underlying soybean leaf abscission**.

Table S1 **Excel file listing expression and annotation of 14,654 differentially expressed genes (DEGs) that were > eight-fold abscission-specific or changed > eight-fold with time and *p* < 0.015**.

Table S2 **Excel file listing expression and annotation of 188 abscission-specific soybean TFs (Figures 1D, 2)**.

Table S3 **Excel file listing expression and annotation of 48 soybean TFs depicted in Figures 3A,B**.

Table S4 **Excel file listing expression and annotation of 520 DEGs depicted in Figures 4A,B**.

Table S5 **Excel file giving additional information on ATRM genes used for transcriptional networks**.
