# Supplementary material for: Transcriptome Analysis of Soybean Leaf Abscission Identifies Transcriptional Regulators of Organ Polarity and Cell Fate
Source: Front Plant Sci. 2016 Feb 17;7:125. doi: 10.3389/fpls.2016.00125 (PMC4756167; doi:10.3389/fpls.2016.00125)
Supplement: Figure S1 — GO term analysis of abscission-specific TF Clusters 3, 4, and 5 (Figures 3A,B). [file Image1.PDF]

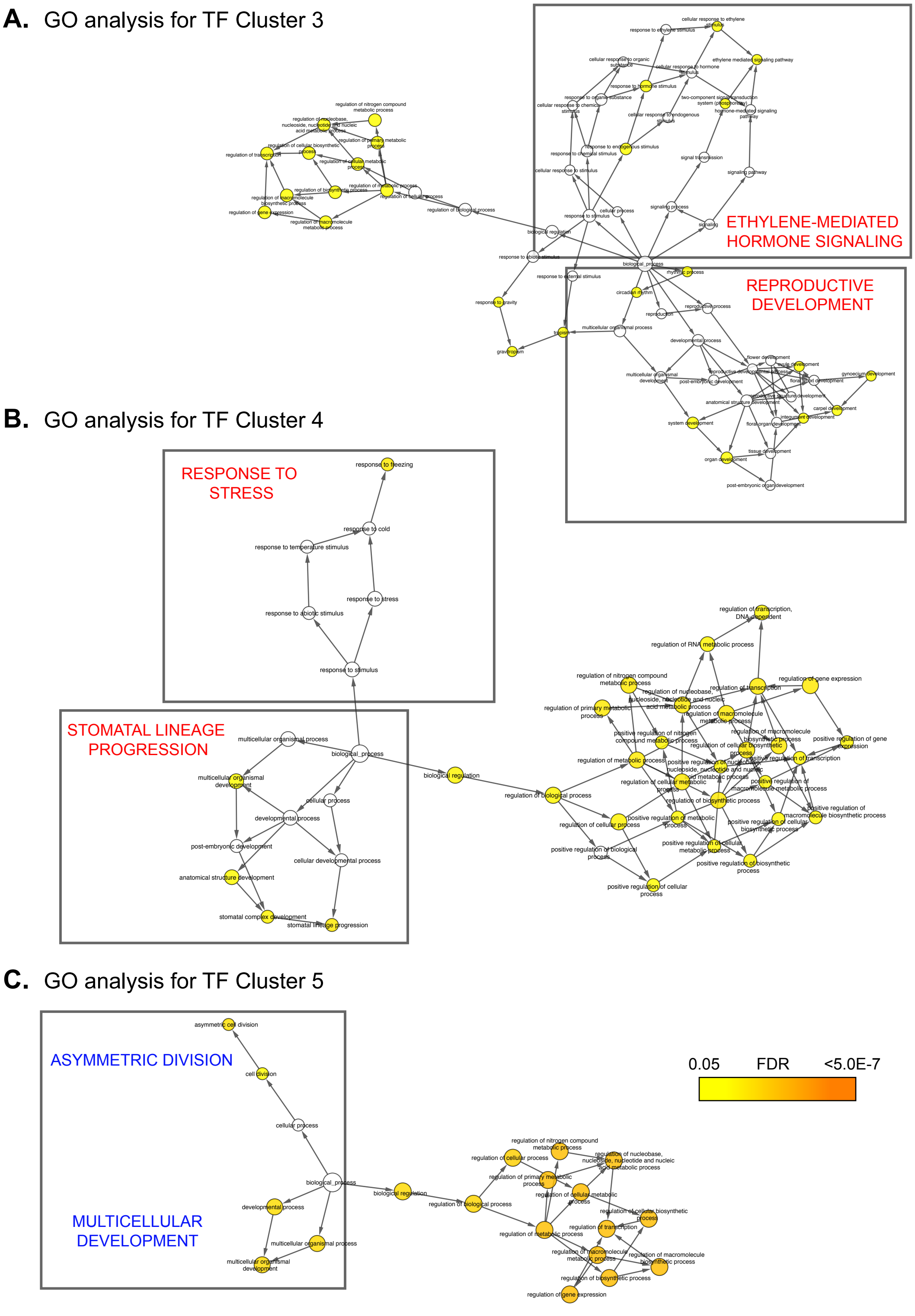

Figure S1. Gene Ontology (GO) term network analysis (BinGO) for TF Clusters 3, 4 and 5 in Figure 3 having 4-fold higher expression in the LAZ/NAZ. Enrichment clusters with similar biological processes are boxed and a summary of the biological process is printed in red or blue inside the box depending on whether the cluster was higher or lower, respectively, in the LAZ. The color bar indicates the range of statistical significance from 0.05 to <5.0 x 10^{-7} for the enrichment of the GO term (colored circles) in the test set (Cluster). *P*-values were adjusted using a Benjamini and Hochberg False Discovery Rate (FDR) correction.
