# Supplementary material for: Transcriptome Analysis of Soybean Leaf Abscission Identifies Transcriptional Regulators of Organ Polarity and Cell Fate
Source: Front Plant Sci. 2016 Feb 17;7:125. doi: 10.3389/fpls.2016.00125 (PMC4756167; doi:10.3389/fpls.2016.00125)
Supplement: Figure S3 — GO term analysis of abscission-specific DEG Clusters 2, 3 and 8 for the entire transcriptome (Figures 4A,B). [file Image3.PDF]

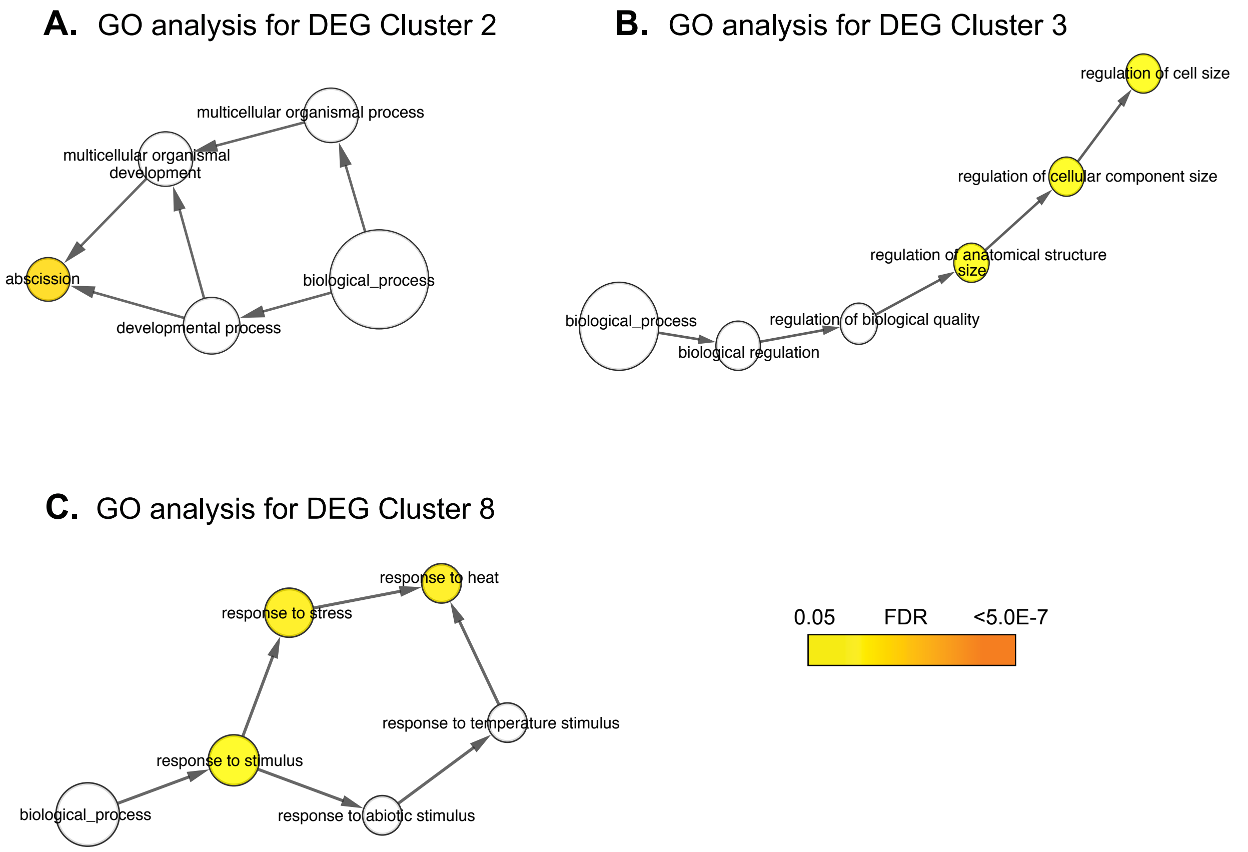

Figure S3. Gene Ontology (GO) term network analysis (BinGO) for DEG Clusters 2, 3 and 8 in Figure 4 having 4-fold higher expression in the LAZ/NAZ. The color bar indicates the range of statistical significance from 0.05 to <5.0 x 10^{-7} for the enrichment of the GO term (colored circles) in the test set (Cluster). *P*-values were adjusted using a Benjamini and Hochberg False Discovery Rate (FDR) correction.
