# Supplementary material for: Transcriptome Analysis of Soybean Leaf Abscission Identifies Transcriptional Regulators of Organ Polarity and Cell Fate
Source: Front Plant Sci. 2016 Feb 17;7:125. doi: 10.3389/fpls.2016.00125 (PMC4756167; doi:10.3389/fpls.2016.00125)
Supplement: Figure S4 — Less extensive transcriptional networks underlying soybean leaf abscission. [file Image4.PDF]

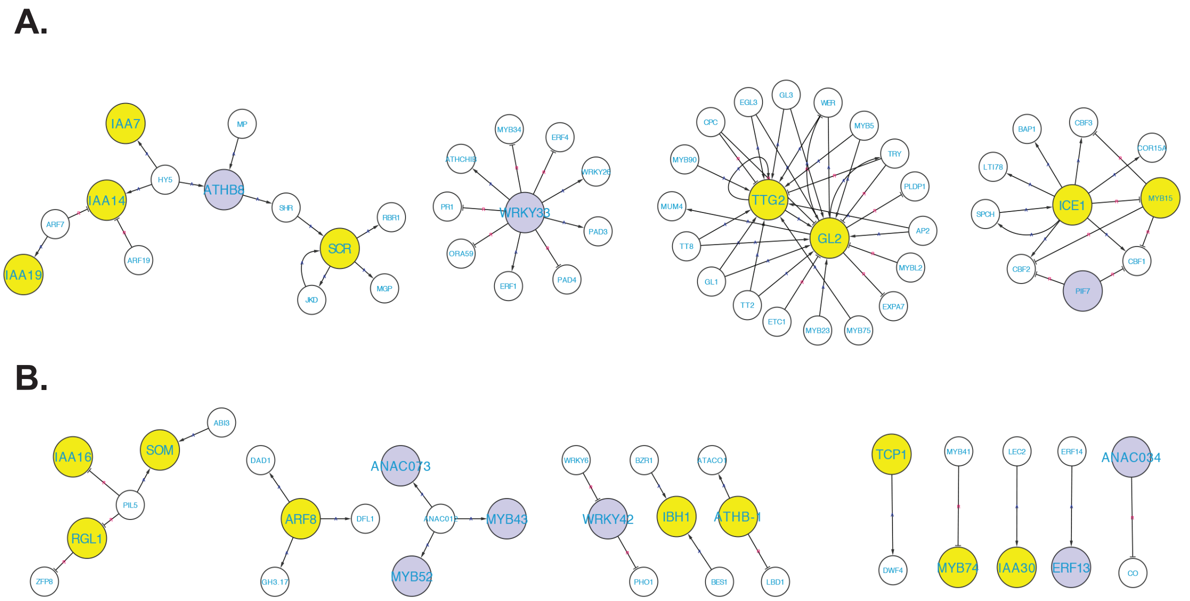

Figure S4. Less extensive transcriptional networks underlying soybean leaf abscission. Networks were generated as described in Figure 5. Yellow inside the circle indicates that the soybean gene was more highly expressed in the AZ and blue inside the circle indicates less strongly expressed in the AZ.
